# Leprosy

**DOI:** 10.4269/ajtmh.13-0668

**Published:** 2014-08-06

**Authors:** Luis A. Marcos, Stephen Conerly, Sue Walker

**Affiliations:** Infectious Diseases, Hattiesburg Clinic, Hattiesburg, Mississippi; Dermatology, Hattiesburg Clinic, Hattiesburg, Mississippi; Pathology Department, Hattiesburg Clinic, Hattiesburg, Mississippi

**Figure 1.**
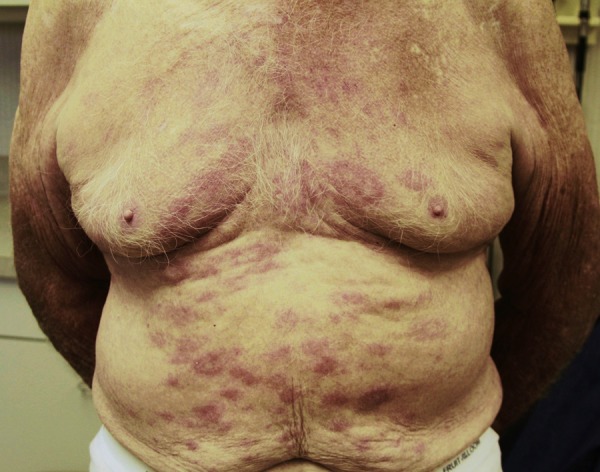
Erythematous rash in trunk.

**Figure 2.**
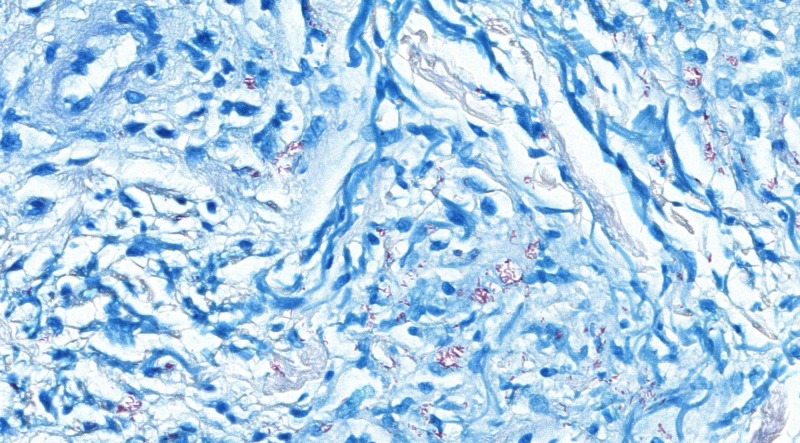
Fite stain-positive for *Mycobacterium* spp. Magnification, ×40.

An 85-year-old man presented with an 8-month history of a pruritic rash on his chest, back, and abdominal areas. The patient was born and raised in Mississippi, where he has lived most of his life. The skin lesions were characterized by being round, erythematous, and macular with slightly raised vague borders and a slight hypopigmentation in some lesions (Figure 1). Skin biopsy revealed diffuse granulomatous infiltrate (not well-formed granulomas), predominantly with foamy histiocytes and numerous acid-fast bacilli (AFBs) observed by the Fite stain (Figure 2). The AFBs were seen in the cutaneous nerves. By the Ridley–Jopling classification system, the diagnosis was consistent with borderline lepromatous leprosy. There was no peripheral nerve enlargement. He was started on clofazimine (50 mg daily), dapsone (100 mg daily), and rifampin (600 mg one time per month) and had remarkable improvement and great tolerance. Leprosy is a chronic infection caused by *Mycobacterium leprae*, an AFB affecting mainly the cooler parts of the body, such as the skin, upper respiratory passages, and peripheral nerves. Anesthesia caused by nerve involvement is a major problem in patients with leprosy, because it may lead to clinical manifestations where the affected peripheral nerves are distributed (i.e., foot drop, clawed hands, ulcers on feet or hands, corneal ulceration, etc.). In the United States, there were approximately 213 new cases reported in 2009.^1^ Contact with armadillos has been shown to be a significant risk factor for acquiring leprosy in humans.^2^ Identical *M. leprae*-genotyped strains have been found in both animals and humans in the United States, showing that these animals play a key role in the transmission of the bacteria to humans.^3^ However, our patient denied any contact with these animals. Physicians and primary care providers should be aware of leprosy in rural areas of the south central United States, and it can be present with a slowly progressive erythematous skin rash, despite denying contact with armadillos.
